# Emerging Roles of Bile Acids and TGR5 in the Central Nervous System: Molecular Functions and Therapeutic Implications

**DOI:** 10.3390/ijms25179279

**Published:** 2024-08-27

**Authors:** Lorenzo Romero-Ramírez, Jörg Mey

**Affiliations:** 1 Laboratorio de Regeneración Neuronal, Hospital Nacional de Parapléjicos, Servicio de Salud de Castilla-La Mancha, 45071 Toledo, Spain; 2 EURON Graduate School of Neuroscience, Maastricht University, 6229 ER Maastricht, The Netherlands; jmey@sescam.jccm.es

**Keywords:** bile acids, cholesterol, INT-777, neuroinflammation, neuronal signaling, neuroprotection, neuropathology, TGR5, TUDCA

## Abstract

Bile acids (BAs) are cholesterol derivatives synthesized in the liver and released into the digestive tract to facilitate lipid uptake during the digestion process. Most of these BAs are reabsorbed and recycled back to the liver. Some of these BAs progress to other tissues through the bloodstream. The presence of BAs in the central nervous system (CNS) has been related to their capacity to cross the blood–brain barrier (BBB) from the systemic circulation. However, the expression of enzymes and receptors involved in their synthesis and signaling, respectively, support the hypothesis that there is an endogenous source of BAs with a specific function in the CNS. Over the last decades, BAs have been tested as treatments for many CNS pathologies, with beneficial effects. Although they were initially reported as neuroprotective substances, they are also known to reduce inflammatory processes. Most of these effects have been related to the activation of the Takeda G protein-coupled receptor 5 (TGR5). This review addresses the new challenges that face BA research for neuroscience, focusing on their molecular functions. We discuss their endogenous and exogenous sources in the CNS, their signaling through the TGR5 receptor, and their mechanisms of action as potential therapeutics for neuropathologies.

## 1. Is the Central Nervous System (CNS) a Source of BAs?

**Figure 1:**
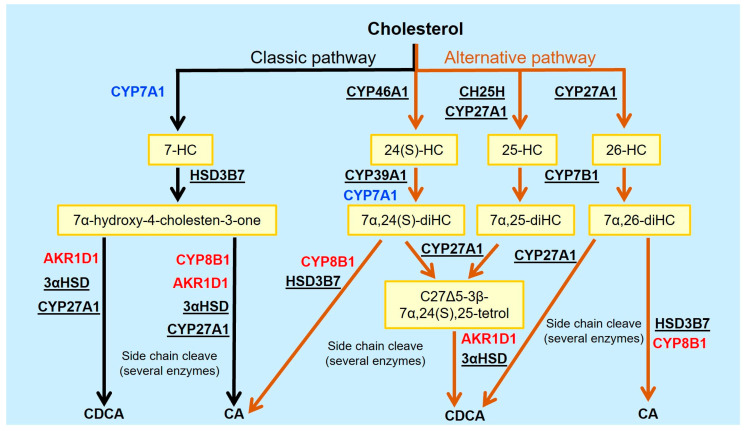
**Bile acid synthesis pathways in the liver and CNS. Some enzymes of bile acid synthesis are not expressed in the CNS.** Enzymes expressed in the CNS are underlined in black. Enzymes with low expression or not expressed in the CNS are in red. The enzymes that are expressed in activated glial cells are in blue.

**Figure 2:**
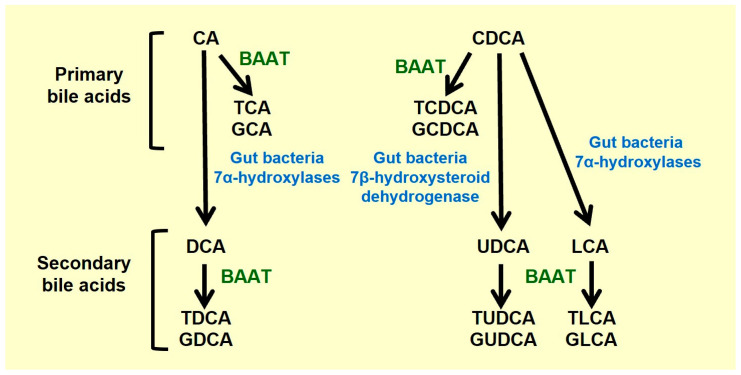
**Biosynthesis of conjugated bile acids in humans. Cholic acid (CA), chenodeoxycholic acid (CDCA) and their conjugates with glycine and taurine are primary bile acids.** CA is transformed by the gut bacteria 7α-hydroxylases into deoxycholic acid (DCA). CDCA is transformed by the gut bacteria 7α-hydroxylases and gut bacteria 7β-hydroxysteroid dehydrogenases into lithocholic acid (LCA) and ursodeoxycholic acid (UDCA), respectively. After the reuptake of these bile acids in the ileum, they might be conjugated in the liver. These bile acids (DCA, LCA and UDCA), together with their conjugated forms, are considered secondary bile acids. BAAT (bile acid-CoA: amino acid N-acyltransferase), GCA (glycocholic acid), GCDCA (glycochenodeoxycholic acid), GDCA (glycodeoxycholic acid), GLCA (glycolithocholic acid), GUDCA (glycoursodeoxycholic acid), TCA (taurocholic acid), TCDCA (taurochenodeoxycholic acid), TDCA (taurodeoxycholic acid), TLCA (taurolithocholic acid), TUDCA (tauroursodeoxycholic acid).

Bile acids are predominantly synthetized in the liver from cholesterol by two different pathways: the classic (or neutral) pathway and the alternative (or acidic) pathway (for more information, see [1,2]) (Figure 1). While both pathways take place in the liver, the alternative pathway can occur in other tissues as well. The bile acids produced in these reactions and their conjugates with glycine or taurine are called primary BAs. Both classic and alternative pathways synthesize cholic acid (CA or 3α,7α,12α-trihydroxy-5β-cholan-24-oic acid) and chenodeoxycholic acid (CDCA or 3α,7α-dihydroxy-5β-cholan-24-oic acid) (Figure 1). These can be conjugated with glycine or taurine, which produces glycocholic acid (GCA), taurocholic acid (TCA), glycochenodeoxycholic acid (GCDCA) and taurochenodeoxycholic acid (TCDCA), respectively (Figure 2). The relative importance of one or the other synthesis pathway depends on the species. In rodents, about 50% of the BAs in the gut are produced by the alternative pathway, whereas less than 10% are in humans.

The conjugation of BAs with taurine or glycine in the liver is catalyzed by the enzyme BA-CoA: amino acid N-acyltransferase (BAAT) (Figure 2), increasing their amphipathic properties. This reduces their toxicity and increases their solubility, thereby facilitating their release into the bile caniculi [3]. While BAs conjugated with glycine are the most abundant in humans [4], taurine conjugation is the most abundant in rodents [5]. 

Once primary BAs are released into the gut, the bacteria from the colon modify them into secondary BAs (Figure 2). Conjugated primary BAs are deconjugated by bacterial hydrolases [6]. This step is essential for the transformation of CA and CDCA into secondary BAs [7]. While CA is modified into deoxycholic acid (DCA or 3α,12α-dihydroxy-5β-cholan-24-oic acid), CDCA is transformed into lithocholic acid (LCA or 3α-hydroxy-5β-cholan-24-oic acid) or ursodeoxycholic acid (UDCA or 3α,7β-dihydroxy-5β-cholan-24-oic acid). Most of the BAs released into the gut (95%) are reabsorbed in the ileum and transported back to the liver through the portal vein [1]. The majority of these BAs are secreted into the bile secretion to be released again into the small intestine, and only a small proportion (<10%) reaches the systemic circulation [1]. 

Depending on the food intake, the plasma concentration of BAs fluctuates between 5 and 15 micromoles in humans [8]. The concentration of BAs in the bloodstream is tightly regulated. A high concentration of BAs (>200 µM) might modify the BBB, increasing its permeability and allowing BAs and other substances to diffuse into the CNS [9]. During some pathological processes, like liver failure, the concentration of BAs in the blood may increase dramatically (>1 mM). This can damage the lipid bilayers of all tissues due to their capacity to dissolve lipids [9,10]. 

More than 40 primary and secondary BAs are found in the CNS [11,12]. Among those isolated from the brain [12], the most abundant are CDCA, DCA and CA [11,12]. Since the enzymes required for the synthesis of secondary BAs (7α-hydroxylases and 7β-hydroxysteroid dehydrogenases) have not been identified in the CNS, secondary BAs can only reach the CNS through the systemic circulation [1]. Unconjugated BAs (e.g., CDCA, DCA and CA) can cross the BBB and enter the CNS by passive diffusion, the efficiency of which depends on their hydrophobicity [13]. Consequently, the concentration of unconjugated BAs in the CNS correlates with their concentration in the systemic circulation [13,14]. As conjugated BAs (e.g., GCDCA) have a larger structure and amphipathic properties, they require active transportation through transmembrane transporters in order to have access to the CNS [15]. Accordingly, the abundance of conjugated bile acids in different areas of the CNS depends on the expression of members of the solute carrier family (e.g., apical sodium-dependent bile acid transporter (ASBT) and organic anion transporting polypeptides or OATP) and the ATP-binding cassette transporters family (e.g., multidrug-resistant protein 2) [16,17,18]. This fact limits their activity in the CNS.

These transporters not only facilitate the access of molecules from the bloodstream, but they also protect the CNS from the accumulation of potentially toxic molecules, transporting them out into the bloodstream.

The CNS contains the highest concentration of cholesterol in the body, representing 15% and 23% of the total amount in mice and humans, respectively [19]. Cholesterol is a fundamental component of cell membranes and nervous connections (e.g., myelin sheaths, synapses and dendrites) and a precursor for several bioactive lipids that are synthesized in the CNS (e.g., neurosteroids). In the CNS, cholesterol is synthesized de novo because the cholesterol from the bloodstream cannot cross the BBB [20]. However, there are some cholesterol derivatives that can pass through the BBB. One example is (25R)26-hydroxycholesterol (26-HC, i.e., cholest-5-ene-3β,27-diol, which is also, wrongly, named 27-hydroxycholesterol [21].

The sterol 24-hydroxylase or cytochrome P450 family 46, subfamily A, member 1 (CYP46A1) is highly expressed by CNS neurons [22,23], also by astrocytes [24], microglia [25] and oligodendrocytes [26]. CYP46A1 transforms cholesterol into 24S-hydroxycholesterol (24S-HC) [23], decreasing the hydrophobicity of cholesterol and facilitating its transportation out of the CNS. This oxysterol can be transformed into BAs through the oxysterol 7-alpha-hydroxylase or cytochrome P450 family 39 subfamily A member 1 (CYP39A1) [27]. Although enzymatic activity of CYP39A1 has been reported in rat brains [28], its expression was detectable in microglia, oligodendrocytes, neurons and astrocytes only at low levels [27,29]. It is commonly accepted that the synthesis of 24-hydroxycholesterol has the physiological function of clearing cholesterol from the CNS [30]. The 24-hydroxycholesterol can finally be transformed into BAs in the liver [27]. 

Neural cells express most of the alternative pathway enzymes required for BA synthesis (Figure 1, Table 1), like sterol 27-hydroxylase or cytochrome P450 family 27 subfamily A member 1 (CYP27A1) [24,31], oxysterol 7α-hydroxylase or cytochrome P450 family 7 subfamily B member 1 (CYP7B1) [31,32,33,34], 3-beta-hydroxy-delta-5-C27-steroid oxidoreductase (HSD3B7) [35,36,37] and 3α-hydroxysteroid dehydrogenase (3αHSD) [38,39,40]. However, some enzymes required in the last steps of BA synthesis are not expressed in the brain (e.g., cytochrome P450 family 8 subfamily B member 1 or CYP8B1) [29] and others, e.g., aldo-keto reductase family 1 member D1 (AKR1D1), are found at very low levels [29,41] (Table 1). Whether this low expression is enough to produce bile acids in a concentration with a meaningful effect requires further investigation. It is also unknown whether other enzymes with similar activities are expressed in the CNS. 

Interestingly, there are some enzymes of the BA synthesis pathways with low gene expression under physiological conditions, like cytochrome P450 family 7 subfamily A member 1 (CYP7A1), that are highly expressed in activated microglia and astrocytes [42]. In fact, CYP7A1 is the only rate-limiting enzyme in the classical pathway. This suggests the possibility that the synthesis of BAs in the CNS changes significantly with the activation of glial cells under pathological conditions. 

Some enzymes of the BA synthesis pathways expressed in the CNS are involved in the synthesis of neurosteroids (e.g., 3αHSD and HSD3B7) [43]. Others take part in the oxidation of cholesterol, resulting in hydroxycholesterol metabolites or oxysterols. CYP27A1 is involved in the CNS cholesterol clearance through a CYP46A1-independent pathway [44]. CYP27A1 can catalyze the conversion of cholesterol to 26-HC [45,46,47]. 

Furthermore, CYP27A1 can synthesize the oxysterol 25-hydroxycholesterol (25-HC) [46]. Activated microglia [36,48] and macrophages [49,50] can also produce 25-HC through the activation of a specific cholesterol-25-hydroxylase (Ch25H). The subsequent hydroxylation of 25-HC by the CYP7B1 enzyme produces 7α,25-dihydroxycholesterol (7,25-dHC) [51]. All of these oxysterols are BA precursors (Figure 1). Oxysterols themselves have biological functions, as they regulate lipid metabolism, inflammation and the innate and adaptative immune responses [49]. The oxysterols activate receptors that are involved in cholesterol metabolism, the regulation of inflammasome activity and cell migration [49].

Is the brain a source of bile acid synthesis? Taken together, CYP27A1, CYP46A1 and other enzymes of the alternative pathway expressed in the CNS contribute to oxysterol production, cholesterol clearance and the synthesis of neurosteroids, but whether they are involved in BA synthesis in the CNS remains to be confirmed. Research on these enzymes in specific CNS regions and populations of neural cells might provide better information on this question. 

## 2. Bile Acid Signaling in the Nervous System

Over the last decades, BAs have been reported as neuroprotective and anti-inflammatory substances in many CNS pathologies [52,53,54]. However, BA signaling has also been associated with the detrimental effects of the hepatic encephalopathy caused by liver dysfunction [55]. As a consequence of the accumulation of BAs in the bloodstream, the BBB permeability increases, triggering neuroinflammation and neuronal dysfunction [55]. These contradictory effects involve the activation of different BA receptors.

**Figure 3:**
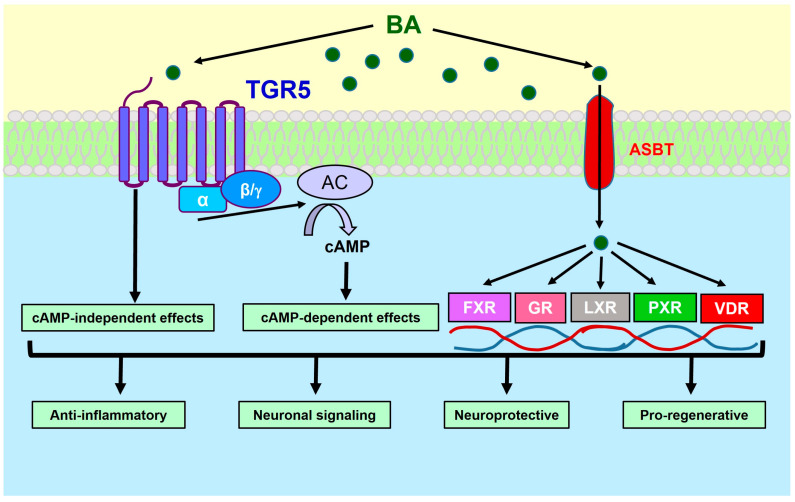
**Bile acid (BA) signaling in the nervous system.** Most effects of BAs in the CNS are mediated via the Takeda G protein-coupled receptor 5 (TGR5), with cAMP as a second messenger, activating protein kinase A (PKA) or nucleotide exchange factors. TGR5 also has PKA-independent effects. Inside the cell, BAs can directly bind to nuclear receptors, such as farnesoid X receptor (FXR), glucocorticoid receptor (GR), liver X receptor (LXR), pregnane X receptor (PXR) and vitamin D receptor (VDR). Conjugated BAs require a transporter (e.g., ASBT) to get into the cells. This figure is a modification of a figure that we published previously [102].

BAs bind and signal to the cells through both transmembrane receptors and intracellular receptors (Figure 3). In the first group, there are Takeda G protein-coupled receptor 5 (TGR5, also named G protein-coupled bile acid receptor 1, GPBAR1) [56] and sphingosine-1-phosphate receptor 2 (S1PR2) [57]. The second group contains farnesoid X receptor (FXR) [58], pregnane X receptor (PXR) [59,60], vitamin D receptor (VDR) [61,62], glucocorticoid receptor (GR) [63] and liver X receptor (LXR) [64,65]. All these receptors are expressed in the CNS [56,57,58,59,60,61,62,63,64,65]. 

Sphingosine-1-phosphate receptor 2 is a G protein-coupled receptor associated with the increase of BBB permeability due to liver dysfunction [57] and stroke [66]. Conjugated bile acids (e.g., TCA [67]) are agonists for this receptor [68]. 

Farnesoid X receptor is a nuclear transcription factor that dimerizes with retinoid X receptor α (RXRα) and binds to FXR response elements in the promoter of their target genes [69]. The transcription factor FXR is also a regulator of BA synthesis [70]; FXR knockout mice show higher serum levels of BAs, which cross the BBB. The animals have impaired recognition memory and motor coordination [71]. Chenodeoxycholic acid and LCA are the most effective agonists for FXR [72]. 

Pregnane X receptor dimerizes with RXRα and regulates the transcription of many enzymes and transporters involved in xenobiotic metabolism [73] and in BA breakdown and degradation [59]. Deletion of PXR in mice severely impairs recognition memory and increases the development of anxiety-like behavior [74]. Lithocholic acid and 3-keto-LCA are the most effective agonists for PXR [59]. 

Vitamin D receptor is another nuclear transcription factor that dimerizes with RXRα. Its functions include the regulation of detoxifying metabolic enzymes that are involved in BA elimination [75,76,77]. Although LCA activates VDR, it is a weak agonist compared to vitamin D [78]. 

Glucocorticoid receptor is a nuclear transcription factor that it is involved in the stress response, learning and memory [79]. Its endogenous agonists are cortisol in humans and corticosterone in rodents [79]. However, TUDCA binds and activates GR [80]. In fact, GR activation has been related to therapeutic effects of TUDCA in rodent models of spinocerebellar ataxia type 3 [81].

Liver X receptors α and β are nuclear transcription factors that heterodimerize with RXRα [82]. Liver X receptor β is ubiquitously expressed, including in the CNS [65]. As macrophages express LXRα, it is likely that microglial cells, the CNS resident macrophages, express it too [83]. In addition, there is evidence that astrocytes might express LXRα [84]. Liver X receptor signaling links lipid metabolism to inflammation and the immune system [85]. The oxysterols are the endogenous agonists for LXR [82]. Hyodeoxycholic acid is a weak agonist for LXR [86]. 

Most studies on the role of BAs in the CNS focus on TGR5. Outside the nervous system, it is highly expressed in the digestive tract, liver, gallbladder and especially in the spleen [87]. The expression of the TGR5 transcript is very high in monocytes/macrophages [87].

Neurosteroids are also TGR5 agonists [88,89]. These cholesterol derivatives are synthesized in the adrenal gland, gonads and placenta and reach the CNS via the systemic circulation. In addition, there is an endogenous synthesis of steroids in the CNS [90]. Both steroids that reach the CNS from the circulation as well as those synthesized within it are considered neurosteroids (for more information, [91]). The synthesis of BAs and neurosteroids share common enzymes (e.g., 3αHSD and HSD3B7) [1,43]. Similar to BAs [56,92], neurosteroids have shown neuroprotective and anti-inflammatory activity [91]. The neurosteroid 5β-pregnan-3α-ol-20-one is a more potent activator of TGR5 than the strongest BA agonists (LCA and its conjugates). In addition, pregnandiol and 5α-pregnandione (or allopregnanedione) have similar activity to LCA and its conjugates [93]. Consequently, it has been suggested that these are the endogenous TGR5 agonists in the CNS [48].

### 2.1. Molecular Signaling of TGR5

The presence of enzymes that synthesize BAs and of BA receptors within the CNS suggest that BAs play a role in brain functions. As we learn more about the influence of intestinal microbiota on the gut–brain axis, the central effects of BAs gain additional interest because peripherally produced secondary BAs can reach the brain. It is, therefore, important to understand the molecular signaling of BAs within the nervous system.

#### 2.1.1. TGR5 Expression in the CNS

In the nervous system in general, the expression of the TGR5 transcript is low. However, there are moderate levels of TGR5 in the cerebral cortex and high levels in the hypothalamus and pituitary gland [87]. The BA receptor has been detected in neurons [88], astrocytes [88], microglia [56], Schwann cells [94] and brain endothelial cells [95]. Neurons with TGR5 were reported in the enteric nervous system [96], brain cortex [88], dorsal root ganglia (DRG) and spinal cord [97], hippocampus [98] and hypothalamus [99]. 

The strongest endogenous agonists for TGR5 are taurolithocholic acid (TLCA), lithocholic acid (LCA), DCA, CDCA and tauroursodeoxycholic acid (TUDCA) [87]. Other agonists of TGR5 are neurosteroids [88], natural terpenoids (e.g., betulinic acid and oleanolic acid) [100], some antibiotics (e.g., ciprofloxacin) [101] and synthetic compounds like INT-777 [89] and RG-239 [100].

#### 2.1.2. The cAMP Pathway

TGR5 signaling was initially discovered as an inhibitory pathway of lipopolysaccharide-induced responses in macrophages [87]. After ligand binding, TGR5 couples to the Gsα subunit of a heterotrimeric G protein. This activates an adenylyl cyclase (AC), which converts ATP to cAMP, which in turn acts as a second messenger [56,87]. The Gsα/AC-regulated synthesis of cAMP is a classical signaling pathway that TGR5 has in common with other heptahelical receptors, e.g., metabotropic neurotransmitter receptors (Figure 3). Molecular events downstream of cAMP are (1) activation of PKA, which phosphorylates different targets, including the cAMP response element binding protein, thereby activating gene transcription, and (2) activation of phospholipase C via the exchange protein activated by cAMP, which initiates diacylglycerol and inositol-3 phosphate signaling and raises the intracellular concentration of Ca^2+^. While these mechanisms were shown to mediate metabolic and immunologic effects of TGR5 ligands [69], it remains to be investigated whether they are activated by BAs in the brain. 

The cAMP signaling is involved in the anti-inflammatory and cytoprotective effects of TGR5 [56,87], which have been observed when studying neuropathologies. An important downstream target is the transcription factor NFκB, which is a central regulator of inflammation [103,104,105]. While this is inhibited by cAMP [106], PKA is also required for complete activation of NFκB signaling [107,108]. The involvement of TGR5 in inflammatory processes will be discussed below. In the presence of lipopolysaccharide (LPS), TUDCA also increased the activation of the transforming growth factor β (TGFβ) pathway through the induction of TGFβ2 and TGFβ3 [109]. We will address these mechanisms below when discussing the role of BAs in neuropathology.

As mentioned above, BAs can directly bind to nuclear receptors and thereby affect transcriptional and epigenetic regulation, but there is yet little evidence for their involvement in brain physiology. Detailed information and additional literature on these pathways are available in the review by Fleishman and Kumar (2024) [69].

#### 2.1.3. Neuronal Excitability

Independent of the effect on NFκB, TGR5 is involved in the regulation of neuronal activity. A recent study with cell-specific deletion of TGR5 demonstrated that the BA receptor regulated the excitability of GABAergic neurons in the lateral ganglionic eminence, which affected calcium calmodulin kinase IIα (CaMKIIα)-expressing neurons in the dorsal CA3 region of the hippocampus [110]. In this case, the molecular mechanism of TGR5 was mediated via phosphorylation of extracellular signal-related kinase (ERK) but not PKA or AK strain transforming (AKT) [110]. As it was found in gastric carcinomas, BA/TGR5 signaling may cause phosphorylation of ERK by the activation of the epidermal growth factor receptor [111]. Direct effects of TGR5 on the activity of CaMKIIα-expressing neurons in the dentate gyrus did implicate PKA, but this worked via phosphorylation of the catalytic unit rather than the conventional cAMP mechanism [110].

Poole and colleagues (2010) showed that TGR5 was expressed in the myenteric neurons of the intestine [96]. The BAs released to the duodenum might be responsible for the activation of TGR5 receptors in the inhibitory motoneurons that slow down transit in the intestine to permit complete digestion and absorption of nutrients [96]. Interestingly, postprandial BAs transported by the systemic circulation into CNS may be responsible for satiety via the activation of TGR5 in the hypothalamic neurons of the arcuate nucleus [99]. These neurons regulated the expression of agouti-related peptide/neuropeptide Y, promoting hunger signals, via TGR5 activity: administration of a BA mix or of the TGR5 agonist INT-777 into the CNS caused an anorexia-like state in wild-type mice. Conversely, deletion of agouti-related peptide or of TGR5 in neurons increased food intake [99]. In conclusion, there is increasing evidence that TGR5 in nerve cells elicits physiological effects via mechanisms that do not involve the traditional Gs/AC/cAMP/PKA pathway.

### 2.2. Putative Physiological Functions of TGR5 in the Brain 

Transgenic mice with deletion of the TGR5 receptors were created almost two decades ago [112,113]. Homozygous TGR5 knockout animals had an altered BA metabolism, and increased fat accumulation when given a high-fat diet, but essentially revealed no pathological phenotype. In these and subsequent studies, neurological functions were not investigated in detail [114]. While it thus appears that TGR5 is not important for brain development, the receptor may still have a role in neuronal functions under certain physiological conditions.

#### 2.2.1. Sensory Signaling

As a result of a study to find out the origin of the pruritus and painless jaundice that patients with cholestatic liver disease suffer, Alemi and colleagues found that TGR5 was expressed in the peptidergic neurons of the spinal cord and dorsal root ganglia (DRG) that transmit itch and pain [97]. While the scratching and analgesia were attenuated in TGR5 knockout mice compared with the wild type, the scratching was exacerbated in TGR5-overexpressing mice [97]. It was proposed that TGR5 could have been activated by bile acids arriving from the bloodstream or by endogenous bile acids synthesized in the CNS. As a third possible explanation, it was proposed that the endogenous TGR5 agonists are neurosteroids [97]. In fact, neurosteroids were found to be generated in DRG, the dorsal horn of the spinal cord, nociceptive supraspinal nuclei and the somatosensory cortex, some of which are specifically involved in nociception [115]. Neurosteroids are modulators of GABAergic signal transduction [116]. 

#### 2.2.2. Mood and Memory

Very little is known about the role of TGR5 in higher brain functions. As discussed below, BA effects were found in animal models of depression and neurodegenerative disorders. These intervention studies raise the possibility that TGR5 is also involved in the regulation of these brain functions under non-pathological conditions. For instance, dysfunction of TGR5 signaling has been implicated in depressive-like behavior in male mice [98] after chronic social defeat stress or chronic restrain stress. The expression of TGR5 is reduced after chronic stress exposure [110]. In transgenic mouse models of Alzheimer’s disease, beneficial [117] as well as detrimental effects [118] of TGR5 activation were found on memory performance.

#### 2.2.3. Apoptosis and Microglia Activity

In development but also in the adult brain, programmed cell death, synaptic stripping and phagocytosis are physiological processes. Since BA effects on apoptosis and the phagocytosis activity of microglial cells are well documented, TGR5 may be involved in their regulation. The initial interest in the use of TGR5 agonists in neuropathologies was due to their neuroprotective effects (e.g., TUDCA [119,120] and INT-777 [121]). Castro and colleagues (2004) found that TUDCA reduced glutamate-induced apoptosis in cortical neurons [120]. The treatment with TUDCA increased the activation of PI-3-kinase, reducing the translocation of the pro-apoptotic protein Bcl being B-cell lymphoma-2 (Bcl-2)-associated agonist of cell death (BAD) to the mitochondria [120]. The synthetic TGR5 agonist INT-777 reduced oxidative stress and apoptosis of neurons after subarachnoid hemorrhage [122]. Treatment increased the expression of the pro-survival protein Bcl-2, aldehyde dehydrogenase 2 (ALDH2) and heme oxigenase-1, while reducing the expression of BAD, cleaved caspase-3 and 4-hydroxinonenal, a marker of oxidative stress. The neuroprotective effect of INT-777 was partially mediated by the cAMP/protein kinase Cε/ALDH2 pathway [122]. Molecular mechanisms will be discussed with the evidence for cytoprotective effects of BAs in diseases.

A different aspect with relevance for tissue homeostasis is the effect of BAs on microglia. TUDCA reduced the migratory capacity of microglial cells treated with IFNγ [123] and restored the phagocytic activity when it was compromised by proinflammatory stimuli [124,125]. TLCA partially restored myelin phagocytosis inhibited in proinflammatory macrophages in a PKA-dependent manner [126]. These effects of TGR5 agonists were studied in the context of inflammation and involved inhibition of the NFκB pathway [104,127]. It will be interesting to investigate whether BA effects on microglia are also relevant for non-pathological processes such as synaptic stripping.

## 3. Bile Acids in Neuropathologies

The first studies using BAs in animal models of neuropathologies were published two decades ago [119,128,129]. After this, translational research with human patients began quickly, and by now, clinical studies have already tested BAs in amyotrophic lateral sclerosis (ALS), Parkinson’s disease (PD), Huntington’s disease (HD) and Alzheimer’s disease (AD; reviewed in [52,53,54]). Here we will look at three aspects regarding BAs and neuropathology: (1) To what degree do altered BA profiles have a causative role in the etiology of disease? (2) Does the evidence from animal models and clinical trials support therapeutic treatment with BAs? (3) What are the molecular mechanisms implied in the animal experiments?

### 3.1. Alterations of BA Metabolism in Neurodegenerative Diseases

Significant alterations of the BA spectrum were found in animal models and in clinical investigations of ALS, PD, HD, AD and psychiatric disorders [52,53,118]. Data from feces or serum samples could be linked to the composition of gut microbiota that produces BA metabolites [130]. With increasing age, the synthesis of bile acids decreases in the liver, and serum cholesterol increases, which constitutes a risk factor for health. Due to their physiological effects, it is not only likely that disease-associated BA metabolism is a symptom but also that it influences the progression of some neuropathologies. 

Dietary patterns can increase or reduce the risk and severity of diseases, and one of the reasons for this may be their influence on the serum levels of bile acids [131]. For instance, the so-called Mediterranean diet, with an emphasis on plant-based foods, is associated with a lower risk of AD and less cognitive decline [132]. Whether this and other dietary supplements [133] involve the activation of BA receptors in the brain is an unresolved question.

#### 3.1.1. Amyotrophic Lateral Sclerosis

There is an association between altered BA metabolism and ALS. Transgenic mice with SOD1 mutations, which are used as a model for motor neuron degeneration, have elevated levels of CA, UDCA, ß-muricholic acid (ßMCA), TCA, TCDCA, TDCA, TUDCA, GCA and GDCA in the spinal cord [134]. In ALS patients, serum concentrations of BAs differed from those of healthy subjects. Specifically, UDCA and GUDCA were increased [135]. The diversity of the microbiome is lower in ALS patients [136], and higher cholesterol levels in the cerebrospinal fluid (CSF) of patients may reflect a reduced BA metabolism in the brain [52]. Whether any of this is causally related to ALS is not clear. 

#### 3.1.2. Parkinson’s Disease

Intracerebral injection of α-synuclein fibrils in mice serves as an experimental model for PD. Metabolome analyses of these animals showed alterations in the taurine metabolism of BAs and changing BA profiles in feces and serum, e.g., decreased levels of ωMCA, TUDCA and UDCA. Transgenic overexpression of α-synuclein increased serum levels of several primary and secondary BAs [137]. Plasma levels of CA, CDA, TDCA and GDCA were found to be higher and that of GUDCA to be lower in PD patients than in healthy subjects [138].

#### 3.1.3. Huntington’s Disease

Alterations of BA-related enzyme activities were found in patients as well as in transgenic R6/2 mice, an animal model for HD [139]. An important example is the reduction in CYP27A1, a key enzyme in cholesterol clearance and BA synthesis [140]. A clinical investigation of HD patients found alterations in cholesterol metabolism in the whole body and brain, including reduced synthesis of the bile acid precursor 26-HC [140]. As with other neurodegenerative diseases, gut dysbiosis is observed in HD, but no causal relationship has been established [52].

#### 3.1.4. Alzheimer’s Disease

Various transgenic AD mouse models produce defects in cholesterol metabolism and BA synthesis [118,130]. In humans, the connection between altered BA metabolism and AD is robust, such that BAs are discussed as biomarkers for this disease [141]. Serum levels of GCDCA, GDCA, GLCA, LCA and TDCA were elevated in patients [138,142]. Lower concentrations of CA and CDCA correlated with brain atrophy and amyloid deposition in the brain [52,143]. Increased serum levels of LCA, DCA and GDCA, and reduced CA, CDCA and UDCA, were associated with mild cognitive impairment [141]. In general, the ratios of secondary BAs to primary BAs were higher in the brains of AD patients compared to healthy persons and to those with mild cognitive impairment [12,138]. The increase in the DCA/CA ratio reflects the higher enzyme activity of microbiota. High DCA in the brain accelerated neurodegeneration [118], and age-related cognitive decline can be linked to increased conjugated primary BAs in the brain [141]. It has been suggested that the gut microbiome is an important aspect of the pathology [130,144]. For AD, the question arises whether TGR5 activation actually exacerbates disease progression. Expression of the BA receptor is increased in the brains of transgenic mice with AD-like pathology, and neuron-specific deletion of TGR5 ameliorated the amyloid pathology and reduced cognitive impairment in the animals [118].

#### 3.1.5. Depression and Psychiatric Disorders

Correlations between blood levels of certain bile acids and depressive symptoms, as well as differences between depressed and healthy individuals, indicate an involvement of BAs in mood disorders [145]. As difficult as it is to study human depression in rodents, models based on chronic unpredictable mild stress and chronic variable stress are frequently used. In several studies using these paradigms, bile acids were analyzed in plasma, feces and liver. Altogether, fifteen different compounds were identified as possible biomarkers [145]. Clinical investigations found higher serum levels of LCA and GCA and lower levels of CDCA to be associated with self-rated anxiety and depression [146,147]. Since the molecular causes of psychiatric disorders are not understood, it is difficult to interpret these correlations. 

#### 3.1.6. Multiple Sclerosis

With the prominent role of the immune system in MS, it is not unlikely that BAs play some role in this pathology. Indeed, the microbiome is altered in MS patients, and at least six bacterial genera that are involved in BA metabolism are depleted or enriched in patients with MS [148]. A metabolomics plasma analysis of MS patients found significantly lower levels of primary and secondary BAs in primary progressive MS patients and of secondary BAs also in relapsing-remitting MS [149]. Elevated levels of 25-HC in the CSF of MS patients may indicate elevated cholesterol metabolism, which was also seen in PD and AD [52]. In a recent clinical trial, higher primary BA serum levels at baseline appeared to be beneficial for MS patients as they predicted slower brain and retinal atrophy [150].

### 3.2. Therapeutic Efficacy of BA Treatment in Neuropathology

The cytoprotective and anti-inflammatory effects of BAs make them interesting for therapeutic applications. Animal research has been done for many neuropathologies. With few exceptions, the publications report successful outcomes. However, clinical studies with BAs have not produced a disease-modifying therapy for any neurodegenerative disease by now (Table 2). 

#### 3.2.1. Spinal Cord Injury

Since 2008, thirteen animal studies with bile acids in SCI have been published [102,151]. Despite positive effects on the recovery of sensory–motor functions in the subacute phase, no lasting improvements with TUDCA treatment were found after SCI. Combinatorial treatment of TUDCA with stem cell injection failed to improve the effect of the cellular treatment in one study [152]. In another, the combination of TUDCA and bone marrow-derived stem cells (BMSCs) but not the BA alone was significant compared to SCI controls [151]. Our conclusion is that the reported effects, at least with TUDCA alone, do not support clinical trials. On the other hand, the cytoprotective and anti-inflammatory effects of TUDCA were found in several experiments, suggesting that bile acids can be useful in combinatorial treatments [102,151,152,153]. In some SCI studies, reduced scar formation and better axonal regeneration were observed, though it is not clear whether these were direct effects of TGR5 in astrocytes and neurons [154,155,156].

#### 3.2.2. Amyotrophic Lateral Sclerosis

In the case of motor neuron degeneration, a fast transition was made from basic research to clinical trials without much evidence of the efficacy in animal research. In a study with transgenic SOD1 mice, TUDCA reduced muscle denervation, but clinical symptoms were not evaluated [157]. Prior to this, a number of clinical trials using bile acids in ALS had already started, and some have been completed (Table 2). The first studies did not reveal any clinical benefits [158,159]. A subsequent trial with oral administration of TUDCA combined with phenylbutyrate resulted in a median survival of 25 months of the treated ALS patients, compared to 18.5 months in the placebo group, a significant difference [160]. Recent experiments confirm that TUDCA treatment also slows disease progression but does not stop it [54,161]. Several clinical trials have been performed with AMX0035, a mixture of TUDCA and phenylbutyrate: NCT04987671, NCT03127514, NCT03488524, NCT05286372 and NCT04516096. Despite small effects on disease progression and survivability, this drug has been approved for the clinic [162]. While incremental benefits are expected, the evidence does not suggest that BAs can fundamentally alter the disease progress of ALS. 

#### 3.2.3. Parkinson’s Disease

The degeneration of dopaminergic neurons in the substantia nigra is a prominent observation in PD. Therefore, several animal models, based on the specific toxicity of 6-hydroxy dopamine or 1-methyl-4-phenyl-1,2,3,6-tetrahydropyridine (MPTP), have been developed to study rescue effects on these dopaminergic neurons. In such experiments, TUDCA was cytoprotective, though clinical symptoms were not evaluated [163,164,165,166,167]. In other studies, some motor symptoms, such as foot dragging, latency in a motor swimming test [166] or deficits in the RotaRod test [168], were reduced with TUDCA treatment of MPTP-treated mice. After completing a phase I safety study (NCT02967250), a placebo-controlled randomized clinical trial (RCT) for PD has recently been completed with UDCA (NCT03840005, [169]). Twenty PD patients were treated for 48 weeks with 30 mg/kg UDCA, and eleven patients were assigned to the placebo group. A sensor-based gait analysis demonstrated minor improvements in some patients, but assessment with the MDS-UPDRS rating scale failed to detect a difference between treatment groups [169]. 

#### 3.2.4. Huntington’s Disease

This disease is the first neurodegenerative pathology where bile acids were tested in animal models, more than twenty years ago [128,129]. Huntington’s disease is caused by expanded CAG trinucleotide repeats in the huntingtin gene, which causes neural degeneration by affecting the mitochondria. A rat model to simulate HD consists of systemic injections of 3-nitropropionic acid, which causes a massive loss of striatal neurons due to inhibition of mitochondrial succinate dehydrogenase and oxidative damage. Chronic treatment of the animals with 50 mg/kg TUDCA per day for one month reduced striatal lesions and prevented sensorimotor deficits as measured with the RotaRod assay [128]. Compared to bile acid treatments in other neuropathologies, this was a strong effect. Surprisingly, these studies had little repercussion during the following two decades, until recently, when a clinical trial with ursodiol was realized (NCT00514774). The aims of the study were to measure BA metabolites in the serum and CSF and to establish the safety of the treatment.

#### 3.2.5. Alzheimer’s Disease and Dementia

The experimental evidence for the treatment effects of BAs in animal models of AD is better than for other neurodegenerative diseases. Recently, Song and colleagues (2024) reviewed eight preclinical studies performed between 2006 and 2021 with transgenic AD mouse models. They reported therapeutic effects on histological outcomes, including Aß deposition and the hyperphosphorylation of tau [117]. Three papers obtained behavioral benefits such as improved memory and cognitive performance [170,171,172]. In a transgenic mouse model with neuron-specific overexpression of mutated human amyloid precursor protein and mutated presenelin 1 (APP/PS1), spatial memory was tested with the Morris water maze. Repeated injections of 500 mg/kg TUDCA every three days for three months resulted in a slightly higher preference for the target quadrant in the test session, which the non-treated APP/PS1 mice did not show. Learning itself was not affected [170]. Effects in this model with dietary supplementation of TUDCA were observed by Lo and colleagues (2013), who also investigated social recognition and passive avoidance. TUDCA significantly improved memory retention in these tests [171]. In the third study with APP/PS1 mice, contextual fear conditioning was used to test memory deficits. Dietary supplementation with TUDCA improved discrimination between a conditioned and a novel context but did not affect auditory fear conditioning [172]. Several of these studies showed that BA treatment reduced the deposition of Aß and neuronal loss in the hippocampus [117,170,171,172]. In aged mice, cognitive performance could be improved by reducing the level of conjugated primary BAs in the brain [173]. While most publications assume a positive role of BAs, specifically TUDCA, one recent study with transgenic 5xFAD mice demonstrated that specific deletion of TGR5 in excitatory neurons attenuated Aß deposition and improved cognitive functions [118].

As in other diseases, the translation to the clinic was made using the more hydrophilic TUDCA. In 2021, Amylyx Pharmaceuticals tested the combination of TUDCA and phenylbutyrate, in a placebo-controlled RCT with volunteers that suffered from mild cognitive impairment or early AD (NCT03533257). The study was designed to evaluate safety, tolerability, drug target engagement, brain atrophy, cognition and psychiatric symptoms over 24 weeks. No effects on cognitive or functional measures were found, though the investigators stated that the study was not designed for that purpose [174]. Given the possibility that TGR5 activation accelerates neuronal degeneration in AD [118], caution is advised regarding future clinical trials.

#### 3.2.6. Clinical Depression

Despite the lack of knowledge regarding the molecular mechanisms of psychiatric disorders, animal experiments with BAs have been conducted [175,176]. An important lead is the link between neuroinflammation and clinical depression [177]. Since TUDCA was effective in a mouse model of LPS-induced systemic inflammation [56], this BA might be beneficial in clinical depression. Cheng and colleagues (2019) used LPS to induce depression-like behavior in mice. Pretreatment with 200 or 400 mg/kg TUDCA once daily for seven days attenuated behavioral symptoms in the tail suspension, forced swim and sucrose preference tests, which are traditionally used to assess the effects of antidepressant drugs in mice. The authors consider TUDCA a potential antidepressant because of its inhibitory effect on inflammation and oxidative stress in the brain [176]. A biologically more relevant way of inducing depression-like symptoms in rodents uses chronic stress. Intraperitoneal injections of 0.5–2.5 mg/kg of ganoderic acid produced similar effects to the tricyclic antidepressant imipramine [178]. Ganoderic acid is a plant-derived triterpenoid with a BA structure, whose effects were mediated by FXR in this case. The other BA receptor, TGR5, was implicated in a study, where the molecular effects of TUDCA treatment were investigated in the chronic unpredictable stress model [175]. A dose of 200 mg/kg (but not 100 mg/kg) had a similar antidepressive effect to fluoxetine, a selective serotonin reuptake inhibitor. TGR5-deficient mice show symptoms that are interpreted as depression in the rodent models [98]. Novel results with a local overexpression of TGR5 in the lateral hypothalamic area indicate that the effects of BAs may result from direct modulation of GABAergic neurons that connect to the hippocampus [110]. In conclusion, BAs had a similar efficacy to established antidepressants in rodent studies. To our knowledge, no clinical trials for depression have yet been started with BAs.

#### 3.2.7. Multiple Sclerosis

In animal experiments, multiple sclerosis is frequently studied by inducing experimental autoimmune encephalomyelitis (EAE). In this model, a myelin protein or peptide is injected together with an adjuvant. This elicits an immune response causing CNS demyelination and clinical symptoms. In one study with mice, a daily gavage of 25 mg/kg or 50 mg/kg TUDCA was administered for 25 days starting 2 days before induction of EAE. Both doses significantly reduced inflammation and demyelination and improved the clinical score [179]. Previously, the anti-inflammatory and therapeutic effects of TUDCA in EAE were shown to be mediated by TGR5 [149]. Based on these encouraging results, an RCT has recently been completed (NCT03423121). Of 47 MS patients, 26 were given 2 g/day TUDCA for a period of 16 weeks. The treatment elevated serum levels of TUDCA (the treatment itself), GUDCA, UDCA, LCA and GLCA, and affected the microbiome but it had no effect on clinical symptoms [150].

#### 3.2.8. Stroke and Cerebral Ischemia

In line with the current interest in the gut microbiome, it is being investigated whether the BA profile relates to the clinical prognosis after stroke [180]. Treatment options have also been explored, and these produced some promising results. Activation of TGR5 with INT-777, when given 24 h after unilateral middle cerebral artery occlusion in rats, significantly reduced infarction volume and improved neurological functions [95]. The protective effects were associated with reduced permeability of the BBB. Experiments by this group revealed some underlying processes, specifically the obligatory activation of TGR5 and the involvement of inflammatory reactions [95,181]. 

In conclusion, most intervention studies with rodent models of neurodegenerative diseases revealed the benefits of TUDCA on the cellular level. Functional outcomes also improved, though in no case was the BA treatment able to restore normal physiological functions. The most promising effects were observed in stress models of depression, where TGR5 activation achieved effects similar to established antidepressants. As with other drugs, the main challenge consists in the translation to the clinic. Phase II clinical trials for ALS, PD and MS have not met expectations. The therapeutic efficacy of GCA and CA in infants, children and adolescents with inborn errors of BA synthesis has also been investigated in clinical studies (NCT1438411, NCT01589523, NCT01115582). For neurodegenerative disorders, more research is needed regarding the causative role of BAs and the effect of dietary patterns on BA signaling in the CNS.

### 3.3. Molecular Mechanisms Associated with BA Effects in Disease Models

In the treatment of neurodegenerative pathologies with BAs, three major effects were identified that are at least partially independent and responsible for most of the reported benefits on the functional level: reduction of neuroinflammation, ER stress and apoptosis [53,104,122]. These three mechanisms seem to account for most of the therapeutic effects of BAs in the nervous system. In neurodegenerative diseases where the accumulation of misfolded proteins plays a role, the property of BAs as chaperones is discussed [182]. As mentioned above, the direct effects of TGR5 on neuronal activity may play a role in depression [110]. The regulation of lipid and glucose metabolism by BAs is likely to influence the CNS [130,138], but will not be discussed here. 

#### 3.3.1. Anti-Inflammatory Effect via Production of cAMP

The inhibitory effects of BAs on inflammatory processes have been investigated intensely [56,103,104,183]. As mentioned above, the BA mechanism hinges upon the activation of PKA via TGR5/Gsα and AC and the elevation of intracellular cAMP. This pathway, with the subsequent inhibition of NFκB and the NLRP3 inflammasome, is probably responsible for the inhibition of inflammatory cytokines in almost all pathologies where this was observed [103,183]. In addition, bile acids reduce the transcript expression of pyruvate kinase M2 (PKM2) in microglial cells [184]. The induction of PKM2 activity as a transcription factor induces the expression of the lactate dehydrogenase A, increasing lactate production, which finally activates the inflammasome and the IL-1β production as a consequence [185,186]. An alternative inhibitory mechanism of TGR5 on the inflammasome is suggested by experimental results, which indicate that the specific TGR5 agonist INT-777 induced the interaction between TGR5 and pellino3, an E3 ubiquitin ligase, thereby reducing the activity of caspase-8/NRLP3 and the production of IL-1β [181]. Transforming growth factor β reduces as required for TUDCA-induced reduction in microglial activation [109]. Furthermore, TUDCA reduced the migratory capacity of microglial cells treated with IFNγ [123] and also the expression of chemokines, e.g., monocyte chemoattractant protein-1, and vascular adhesion proteins required for the infiltration of blood monocytes into the CNS [123]. In most animal experiments, the relatively hydrophilic TUDCA has been used (see above, Section 2.2). 

Several studies corroborate the anti-inflammatory activity of BAs but engage other signaling pathways, independent of TGR5 and the synthesis of cAMP. Bao et al. (2021) demonstrated that the anti-inflammatory effects of ganoderic acid were mediated through FXR and the NLRP3 inflammasome, and that this affected synaptic activity and reduced depressive symptoms [178]. Experiments with the MPTP mouse model of PD identified the nuclear factor erythroid 2 related factor (Nrf2) as a cytoprotective mechanism of TUDCA involving AKT and c-Jun N-terminal kinases signaling [165]. Additionally, TUDCA also increased the activation of the TGFβ pathway through the induction of TGFβ2 and TGFβ3 in an animal model of acute neuroinflammation [109]. The activation of the TGFβ receptor was required for TUDCA-induced reduction of microglia/macrophage activation in an animal model of acute neuroinflammation [109]. This effect of TUDCA might be due to the inhibition of the expression and release of inflammatory mediators exerted by the activation of the TGFβ pathway [187,188]. 

#### 3.3.2. Endoplasmic Reticulum Stress

Pathological disruptions of the cell’s metabolic homeostasis affect the ER via the activation of membrane proteins PERK, ATF6 and IRE1. These trigger the unfolded protein response (UPR), which, while being a protective mechanism, can cause cell cycle arrest and apoptosis. The UPR is considered a pathological factor in neurodegenerative disorders [189] and can be mitigated by BAs [190,191].

The reduction of ER stress by BAs was addressed in studies of many neuropathologies, including SCI [192], ALS [157], PD [164] and AD [191,193]. In a screening of cytoprotective compounds, ER stress was induced in cell cultures with cyclopiazonic acid. This approach identified TUDCA as a protective influence for motor neurons [157]. The group then corroborated the results in a transgenic SOD1 mouse model of ALS. At this point, we are not able to pinpoint the exact molecular step in the UPR where BAs interfere.

#### 3.3.3. Reduction in Apoptosis and Mitochondrial Dysfunction

Since chronic inflammation contributes to cellular degeneration, the anti-inflammatory effects of BAs are also neuroprotective. Apoptosis, as distinguished from necrotic cell death, is usually measured with staining of activated caspase-3, TUNEL, or DNA fragmentation. Following Khalaf and colleagues [53], we distinguish five main mechanisms of the anti-apoptotic effect of BA: (1) inhibition of the mitochondrial pathway, resulting in lower cytochrome *c* release; (2) inhibition of death receptors and caspase-3 activity; (3) reduction of ER stress, Ca^2+^ release from the ER and caspase-12 activity; (4) modulation of survival signaling pathways; and (5) effects on the expression of genes that are involved in cell proliferation and apoptosis.

In a study on AD, TUDCA affected apoptosis by interfering with p53-mediated processes [194]. Following lesions of the mesostriatal dopamine system with 6-hydroxydopamine, TUDCA reduced apoptosis of cell transplants in the striatum of treated rats [163]. Inhibition of apoptosis after SCI was reported in some cases [151,195], but not in others [152]. Specific prevention of mitochondrial damage by UDCA was shown in the MPTP mouse model, which causes cell death of dopaminergic neurons by inhibiting mitochondrial metabolism. Ursodeoxycholic acid restored the mitochondrial membrane potential, reduced the release of reactive oxygen species and increased ATP levels [168]. In another study with MPTP, TUDCA decreased ROS production and prevented neurodegeneration [164]. It is important to note that while the more hydrophilic BAs are cytoprotective, hydrophobic BAs such as GCA, GDCA and GCDCA promote apoptotic cell death [53].

#### 3.3.4. Bile Acids as Chaperones

Bile acids can act as chaperones [196]. This ability to affect protein folding may account for some cytoprotective effects in neurodegenerative diseases that are associated with the accumulation of misfolded proteins. One cause of ER stress and subsequent apoptosis in SCI is misfolded proteins within the cells [192]. Inhibition of the UPR and tau phosphorylation in neuroblastoma cells by TUDCA has been linked to its property as a chemical chaperone [193]. This mechanism would also be relevant in prion diseases [197,198], as these have their origin in conformational changes of the prion protein (PrP). However, one study with prion-infected mice demonstrated that neither TUDCA nor UDCA were neuroprotective [199]. Cortez and coworkers (2015) found that these BAs reduced aggregation of recombinant PrP without direct interaction with the monomeric protein [198]. 

Research on PD produced some promising data on BAs as chaperones. In PD patients, the aggregation of an insoluble form of α-synuclein in Lewy bodies is causally linked to the pathology. With this target, a library of more than ten thousand derivatives based on bile acid scaffolds was analyzed to find structures that prevent misfolding of α-synuclein. Subsequent simulations showed that TUDCA derivatives interacted best with α-synuclein fibrils [182]. 

In the case of AD, TUDCA treatment reduced two histopathological hallmarks, i.e., neurofibrillary tangles of hyperphosphorylated tau and the accumulation of Aß plaques [117,193]. As attractive as it is to consider direct interference with protein folding [190,191,196], there is little evidence to support this hypothesis in AD. Instead, TUDCA indirectly inhibited γ-secretase, a crucial enzyme in the conversion of APP to Aβ [171]. In vitro, TUDCA did not alter the aggregation Aß peptides [200]. While i.p. administration of TUDCA reduced Aß accumulation in the brain, intraventricular injections did not [191]. Therefore, it remains hypothetical whether BAs influence neurodegenerative diseases by means of their function as chaperones.

#### 3.3.5. Physiology of Nerve Cells

Many types of neurons express TGR5, and since its activation by BAs initiates cAMP signaling, it is to be expected that this would affect neural plasticity and synaptic functions. Effects of TUDCA on dendritic spines, synaptic proteins and electrophysiological responses were demonstrated in primary cultures of hippocampal neurons [201]. It is possible that direct effects on neurons account for some treatment results in vivo. A specific benefit after traumatic lesions was the reduction of neuropathic pain. Intrathecal injection of TGR5 and FXR agonists alleviated mechanical allodynia that was caused by sciatic nerve injury. The molecular mechanism, in this case, seems to involve active GABA_A_R chloride channels [42]. As discussed above (Section 2.2), more evidence of BA interaction with the physiology of neurons was found in animal models of clinical depression. While TGR5 expression levels and TGR5 agonist INT-777 affected neuronal physiology, this implied structural alterations, e.g., altered expression of Kv4.2 voltage-gated potassium channels and spine densities in the dentate gyrus [110]. Effects of TUDCA on synaptic function and plasticity were also investigated in AD animal models [170,172,202]. In these cases, it is more difficult to disentangle the effects from anti-inflammatory and cytoprotective mechanisms.

## 4. Conclusions

It is commonly accepted that bile acids reach the CNS mainly by systemic circulation. Whether there is an endogenous synthesis of bile acids in the CNS is under investigation. Many enzymes of bile acid synthesis are expressed in the CNS, but it is thought that they are involved in the synthesis of oxysterols and neurosteroids, and in cholesterol clearance. Transgenic mice with deletion of the bile acid receptor TGR5 show no apparent pathological phenotype.

TGR5 signaling was initially related to the inhibition of neuroinflammation. Recent reports have discovered roles of TGR5 signaling in several CNS disorders, including neurodegenerative diseases, anorexia and depression. Treatment with BAs frequently was cytoprotective and reduced inflammatory signaling not only in cell culture but also in animal models of SCI, ALS and other human diseases. Neurosteroids were proposed as endogenous TGR5 agonists based on their abundance in the CNS, their affinity for TGR5 and their effects on cytoprotection, inflammation and neuronal signaling.

**Figure 4:**
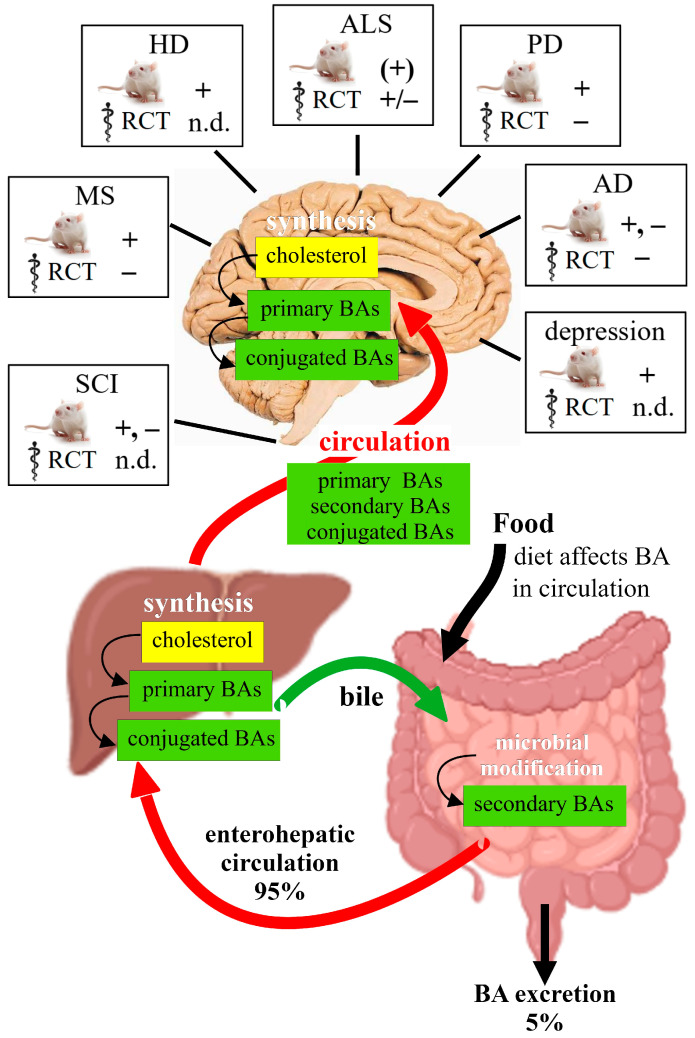
**Synthesis and circulation of BAs.** Primary bile acids are synthesized and conjugated in the liver and released into the duodenum, where they are modified by the microbiome (secondary BAs). Primary, secondary and conjugated BAs reach the CNS via the circulation. Dietary patterns influence the serum levels of BAs. Cholesterol, which does not cross the blood–brain barrier, is synthesized in the CNS, where BA synthesis and conjugation may occur, but this needs further confirmation. The present state of intervention studies with animal models and in randomized clinical trials (RCTs) is indicated for the neuropathologies discussed in this review; + denotes a positive effect and − the absence of effect on functional outcomes; n.d. indicates that no efficacy data are available yet.

Since TGR5 agonists have shown beneficial effects in animal models for neuropathologies, several clinical trials have already been conducted. Treatment with bile acids was generally safe, but clinical studies have not met expectations with respect to efficacy (Figure 4). Regarding the molecular mechanisms of TGR5 activation, the cytoprotective and anti-inflammatory effects have been investigated extensively. We consider it critical to compare the efficacy and side effects of BA treatments with established anti-inflammatory drugs in animal experiments. If these produce favorable results, further clinical trials are indicated. Another emerging field of research is the effect of diet on BA composition in serum and its relevance for CNS functioning and disease. 

## Figures and Tables

**Table 1:** Expression of the enzymes involved in bile acid synthesis in the CNS, neurons, astrocytes, microglia, oligodendrocytes and Schwann cells (SC). The CNS column refers to the transcriptional expression of the enzymes in the Human Protein Atlas (29). The symbol “**+**” means that the expression is reported. The symbol “**–**” means that the expression is not reported or there is no expression. The numbers in parentheses correspond to the article in the bibliography where the expression is referenced.

Enzyme	CNS	Neuron	Astroc.	Microg.	Oligod.	SC
**CYP7A1**	**low**	**–(42)**	**+(42)**	**+(42)**	**–**	**–**
**CYP7B1**	**+(31–34)**	**+(31, 33)**	**+(31)**	**+(31)**	**+(29)**	**+(34)**
**CYP8B1**	**–**	**–**	**–**	**–**	**–**	**–**
**CH25H**	**low**	**+(29)**	**–**	**+(36, 49)**	**–**	**+(29)**
**CYP271**	**+(24, 31)**	**+(24, 31)**	**+(24, 31)**	**+(31)**	**+(24, 31)**	**+(29)**
**CYP391**	**low**	**+(27, 29)**	**+(27, 29)**	**+(27, 29)**	**+(27, 29)**	**+(29)**
**CYP461**	**+(22, 23)**	**+(22, 23)**	**+(24, 31)**	**+(25)**	**+(26)**	**+(29)**
**AKR1D1**	**Low (41)**	**–**	**–**	**–**	**–**	**–**
**3αHSD**	**+(38–40)**	**–**	**–**	**–**	**–**	**–**
**HSD3B7**	**+(35–37)**	**+(29)**	**+(37)**	**+(36)**	**+(29)**	**+(29)**

**Table 2:** Clinical trials with bile acids as treatment of CNS disorders.

Condition	Bile Acid	Trial Number	St	Title	Ph
**ALS**	TUDCA	NCT00877604	C	Efficacy and tolerability of TUDCA in ALS	2
“	“	NCT05753852	R	BA supplementation in patients	3
“	“	NCT03800524	A	Safety and efficacy of TUDCA as add-on treatment in patients affected by ALS	3
“	TUDCA/phenyl butyrate	NCT04987671	A	Pharmacokinetics and pharmacodynamics study of AMX0035 in patients with ALS	1 + 2
“	“	NCT03127514	C	AMX0035 in patients with ALS	2
“	“	NCT03488524	C	Open label extension of AMX0035 in ALS	2
“	“	NCT05286372	M	An intermediate size expanded-access protocol of AMX0035 for ALS	M
“	“	NCT04516096	C	A compassionate use protocol of AMX0035 for treatment of patients with ALS	2 + 3
“	“	NCT05021536	A	Phase III trial of AMX0035 for ALS treatment	3
“	“	NCT05619783	R	Extension study evaluating the safety and tolerability of AMX0035	3
**PD**	UDCA	NCT02967250	C	Brain bioenergetics in PD and response to repeated oral UDCA treatment	1
“	“	NCT03840005	C	Trial of UDCA for PD: the “UP” study	2
**HD**	Ursodiol	NCT00514774	U	Ursodiol in HD	1
**AD**	TUDCA/phenyl butyrate	NCT03533257	C	Safety and biological activity of AMX0035 for the treatment of AD	2
**MS**	TUDCA	NCT03423121	C	BA supplementation in patients with MS	1 + 2

Abbreviations: St, state of trial: R—recruiting, A—active, C—completed, M—approved for marketing, U—unknown.
